# Bisphosphonates and the risk of dementia in patients with osteoporosis or fragility fracture: A population‐based study in Hong Kong

**DOI:** 10.1002/alz.70503

**Published:** 2025-07-21

**Authors:** Chor‐Wing Sing, Koon‐Ho Chan, Patrick K. C. Chiu, Wallis C. Y. Lau, Xiaowen Zhang, Kathryn C. B. Tan, Ching‐Lung Cheung

**Affiliations:** ^1^ Department of Pharmacology and Pharmacy LKS Faculty of Medicine University of Hong Kong Hong Kong SAR Hong Kong; ^2^ Laboratory of Data Discovery for Health Limited (D(2)4H) Hong Kong SAR Hong Kong; ^3^ Department of Medicine School of Clinical Medicine University of Hong Kong Hong Kong SAR Hong Kong; ^4^ Research Department of Practice and Policy UCL School of Pharmacy London UK; ^5^ Hebrew SeniorLife Boston Massachusetts USA

**Keywords:** Alzheimer's disease, antiresorptives, bisphosphonates, dementia, osteoporosis, pharmacoepidemiology

## Abstract

**INTRODUCTION:**

Emerging evidence suggests neuroprotective effects of bisphosphonates. We aim to investigate whether nitrogen‐containing bisphosphonates (NBPs) could reduce the risk of Alzheimer's disease and related dementia (ADRD).

**METHODS:**

We identified patients aged 60+ with osteoporosis or fragility fracture in 2005–2020 from a healthcare database in Hong Kong. Patients receiving NBPs were 1:1 matched with untreated patients and those receiving other anti‐osteoporosis medications (“non‐NBPs”) by time‐dependent propensity score. Follow‐up was conducted until December 31, 2021. Cause‐specific hazard ratios (HRs) and 95% confidence intervals (CIs) were estimated using the Cox proportional hazard model.

**RESULTS:**

Among 121,492 patients (NBP = 15,654, non‐NBP = 6331), we matched 10,833 pairs for NBPs‐vs‐untreated and 3080 pairs for NBPs‐vs‐non‐NBPs. NBP use was associated with a lower risk of ADRD compared to untreated (HR = 0.84, 95% CI = 0.78–0.90) and non‐NBP (HR = 0.76, 95% CI = 0.66–0.89).

**DISCUSSION:**

NBP use was associated with a lower risk of dementia, suggesting further studies are warranted on its potential to improve cognitive function.

**Highlights:**

- Nitrogen‐containing bisphosphonates (NBPs) are associated with a reduced risk of Alzheimer's disease and related dementia.
- NBPs resulted in an absolute risk reduction of 0.007, 0.018, and 0.021 at 1, 3, and 5 years, respectively. The number needed to treat (NNT) with NBPs at 1, 3, and 5 years were 133, 56, and 48.
- There is potential for repurposing NBPs as a therapeutic agent for Alzheimer's disease.

## 1 BACKGROUND

It was estimated that over 55 million individuals worldwide were living with dementia in 2019, and the number is expected to reach 139 million by 2050.^1^ The treatment of Alzheimer's disease and related dementia (ADRD) is challenging due to the limited availability of effective therapeutic agents. For the past two decades, patients have relied on only two classes of symptomatic drugs, namely cholinesterase inhibitors and memantine, to manage their symptoms. Until recently, lecanemab^2^ and donanemab^3^ received full approval from the U.S. Food and Drug Administration (FDA) for the treatment of early Alzheimer's disease or mild dementia. Despite the breakthrough, concerns persist regarding the safety of the medication.^4^ ^5^ The concept of repurposing existing drugs to find alternative therapeutic agents for ADRD has been proposed.^6^ ^7^ This strategy offers a time‐ and cost‐effective approach that complements traditional drug discovery methods.

Bisphosphonates are antiresorptive agents used to treat postmenopausal osteoporosis and other bone‐associated disorders by slowing down bone loss and preventing fractures. Specifically, nitrogen‐containing bisphosphonates (NBP) target the mevalonate pathway by inhibiting the activity of farnesyl pyrophosphate synthase (FPPS), which limits the synthesis of isoprenoids, namely FPP and geranylgeranyl pyrophosphate (GGPP), and prevents the subsequent prenylation of proteins. This ultimately induces apoptosis in osteoclasts via downstream pathways, reducing bone resorption.^8^ Emerging evidence suggests that isoprenoids and protein prenylation play a role in the development of Alzheimer's disease. Studies have found elevated levels of FPP and GGPP in brain tissue from individuals with Alzheimer's disease compared to normal brain tissue,^9^ ^10^ suggesting a specific dysregulation of isoprenoid metabolism in Alzheimer's disease. Also, a variant of FPPS gene has been associated with increased levels of phosphorylated tau protein.^10^ Furthermore, animal studies have demonstrated the neuroprotective effect of alendronate.^11^ ^12^ Based on these findings, it is hypothesized that drugs targeting the isoprenoid pathway and protein prenylation, such as NBPs, could serve as potential therapeutic agents for ADRD.

This study aimed to investigate whether the use of NBPs including alendronate, ibandronate, risedronate, and zoledronate in patients with osteoporosis or fragility fracture is associated with a reduced risk of ADRD. The study used a population‐based electronic medical record (EMR) database in Hong Kong to identify the cohort, which was subsequently matched by propensity score to study the effectiveness of NBPs in reducing risk of dementia.

## 2 METHODS

This study was a retrospective cohort study approved by the Institutional Review Board of the University of Hong Kong/Hospital Authority Hong Kong West Cluster (UW 21‐301).

### 2.1 Data source

Data for the study were extracted from the Clinical Data Analysis and Reporting System (CDARS) in Hong Kong, which contains de‐identified demographic and clinical information on admission, diagnoses, procedures, prescriptions, and laboratory tests from all public hospitals and outpatient clinics in Hong Kong.^13^ The death information in the database is linked to the Hong Kong Death Registry. The database covers over 80% of hospital admissions in Hong Kong^14^ and has been validated for research purposes.^15^, ^16^, ^17^, ^18^ Specifically, diagnostic coding for fragility fracture has been found to have a high positive predictive value (PPV) of 96.8%, indicating a high data quality.^15^ CDARS has been previously used for conducting real‐world studies on fractures^19^, ^20^, ^21^, ^22^ and dementia^23^ ^24^ in Hong Kong.

Diagnoses in the database are coded using the International Classification of Diseases, 9th Revision (ICD‐9) code (ICD‐10 coding has not yet fully implemented in CDARS to date). Medications are coded using a local drug coding system and categorized according to the British Drug Formulary (BNF). Coding for diagnosis and medications used in this study are presented in STable S1.

### 2.2 Study population

The study cohort included patients aged 60 years or older, who had in‐patient or out‐patient diagnoses of osteoporosis or fragility fracture at the spine, humerus, wrist, and hip between January 1, 2005, and December 31, 2020, with no previous diagnoses of osteoporosis or fragility fracture and no previous prescription of any anti‐osteoporosis medications. The date of visiting the out‐patient clinics (for out‐patient diagnosis) or the date of discharge (for in‐patient diagnosis) was considered the “cohort entry date.” Fractures of the spine, humerus, wrist, and hip are classified as major osteoporotic fractures (or fragility fractures) due to their high prevalence in patients with osteoporosis and their significant clinical impact.^25^ ^26^ Many clinical guidelines for osteoporosis management use the risk of major osteoporosis fractures as a criterion for initiating pharmacological treatment.^27^, ^28^, ^29^ Therefore, this study focuses specifically on fractures of these sites.

To minimize selection bias and/or competing risk of death, patients who met any of the following criteria were excluded: (1) previous diagnosis of cancer; (2) previous diagnosis of dementia due to any cause or medication for treatment of dementia (namely donepezil, rivastigmine, galantamine, and memantine); (3) death on the cohort entry date; (4) a length of stay longer than 60 days for those with in‐patient diagnosis of osteoporosis or fragility fracture. It is worth noting that patients with hip fractures, the most severe fragility fractures, generally stayed in the hospital for less than 60 days, as reported in a previous study.^30^ Therefore, those with a longer length of stay may be too frail to take anti‐osteoporosis medications, resulting in indication bias. We screened the cohort using all available EMRs since 1993 in the database.

### 2.3 Exposure

The exposure of interest was determined from the cohort entry date until Dec 31, 2020. This exposure was defined as a prescription for any NBP, which includes alendronate, ibandronate, risedronate, and zoledronate (“NBP‐exposed”). Two control groups were used in the study: (1) untreated controls, which consisted of patients who did not receive any prescription for anti‐osteoporosis medication (“untreated”); and (2) active controls, which included patients who were prescribed other anti‐osteoporosis medications, specifically denosumab, salcatonin, strontium ranelate, and teriparatide. (“non‐NBP‐exposed”).

### 2.4 Outcome

The outcome of interest was the time to incident ADRD including Alzheimer's disease, vascular dementia, senile dementia, and dementia with an unspecified cause. Dementia resulting from other causes was not considered as an outcome. We conducted an internal validation of the diagnostic coding for ADRD (see Supplementary Method for detailed validation procedures), showing a PPV of 81% (95% confidence interval [CI] 77.1–84.9).

**RESEARCH IN CONTEXT**

1. **Systematic review**: The authors reviewed the literature using PubMed and Embase with keywords (Alzheimer OR dementia) AND (bisphosphonate OR alendronic OR risedronic OR zoledronic OR ibandronic) AND (risk OR associated OR association). Limited studies report on the risk of Alzheimer's disease and related dementia (ADRD) associated with NBP use. However, some animal studies demonstrated the neuroprotective effect of alendronate.
2. **Interpretation**: Our findings suggest the potential for repurposing NBPs as therapeutic agents for ADRD.
3. **Future directions**: Our study proposes additional research to (1) validate the association between NBP use and the risk of ADRD in different populations; (2) further understand the mechanism underlying the neuroprotective effects of NBPs; (3) investigate any potential synergistic effects of NBPs and statins, which also target on mevalonate pathway and have been associated with a lower risk of Alzheimer's disease, on the risk of ADRD.

### 2.5 Time‐dependent propensity score matching

To address potential confounding by non‐randomized treatment allocation, we employed the propensity scores (PS) method to balance the baseline characteristics of the comparison groups.^31^ Given that treatment delay is common in patients with osteoporosis or fragility fractures, there is a possibility of immortal time bias that favors the treatment group.^32^ Thus, we performed a time‐dependent PS matching which has been proven as a superior approach to address the bias.^33^ ^34^

The PS was estimated using the Cox proportional hazard regression model, which regressed time‐to‐exposure on covariates including sex, age, calendar year, fracture type, nursing home residency, medical history, and medications taken in the past 30 days (see STable 2 for a full list of covariates). The PS was derived from the cumulative hazards over time.^33^

We performed the sequential matching algorithm by dividing the time‐to‐exposure period into 1‐month blocks since cohort entry. The matching took place within risk sets *R_t_* where *t* represents the month since cohort entry date (e.g., *t *= 1 represents the first month, *t *= 2 represents the second month, and so on). Each risk set *R_t_* consists of all patients at risk of exposure in month *t*. Patients who initiated NBP during month *t* was then matched 1:1 to a control patient. For NBP‐vs‐Untreated analysis, eligible controls were those who had not initiated any anti‐osteoporosis treatment up to and including month *t*. For NBP‐vs‐non‐NBP analysis, eligible controls were those who initiated a non‐NBP treatment during month *t*. Once successfully matched, both NBP‐exposed patients and their matched controls were excluded from subsequent risk sets (R*_t_*_+1_, *R_t_*_+2_, etc.). This means that matched control patients were no longer eligible for matching even if they initiated NBPs in a later month. This sequential matching process ensured that each patient was matched only once, specifically within the risk set corresponding to their time of treatment initiation.^33^ Matching was based on the PS, calendar year, fracture type, sex, and age using sequential greedy matching without replacement, with a caliper of 0.2 standard deviation (SD) of the PS.^35^ ^36^ Calendar year, fracture type, and sex were matched exactly, while age was matched within a 5‐year range. The quality of matching was assessed by estimating the absolute standardized differences (ASD) in covariates between the exposure groups in the matched cohort. An ASD < 0.1 was considered well‐balanced.^37^ Any covariate with an ASD ≥ 0.1 was further adjusted in subsequent regression analyses.[Fig alz70503-fig-0001]

### 2.6 Follow‐up

Given the slow progression of ADRD, an outcome event that occurred early in the follow‐up period is unlikely to be related to the drug. To address this, we introduced a 6‐month latency period^7^ starting from the date of matching. During this latency period, patients who had been diagnosed with ADRD and their matched pair were excluded from the analysis. Follow‐up started (time zero) at the end of the latency period, specifically 6 months (180 days) after the matching date. The matching date corresponds to the date of the first NBP prescription for the NBP‐exposed group, the date of being matched for the untreated group, and the date of first non‐NBP prescription for the non‐NBP‐exposed group. Since both NBP‐exposed and control patients start follow‐up at the same time point, immortal time bias is minimized. Patients were followed until an outcome event (ADRD), death, or December 31, 2021 (end of data collection), whichever came first. A study design schema is presented in SFigure 1 to clarify the follow‐up period.

### 2.7 Statistical analysis

Continuous variables were presented as mean ± SD, and categorical variables as frequency and percentage. Time‐to‐event analysis was used to study the association between the use of NBPs and the risk of ADRD. To address the competing risk of death, cause‐specific hazard ratios (HRs) and 95% CIs were estimated using a Cox proportional hazard model. A robust variance estimator was used in this model to account for the matched nature of the cohort.^38^ Cumulative incidence function (CIF) was used to estimate the cumulative incidence of ADRD in the presence of competing risk. Furthermore, a clustered Fine‐Gray model was employed to test the equality of CIFs between the exposure groups within the matched cohort.^39^ The absolute risk differences (ARDs) between the exposure groups at 1, 3, and 5 years were derived by subtracting the CIFs of the control group from the CIFs of the NBP‐exposed group.^39^ The number needed to treat (NNT) was calculated as the reciprocal of ARD, providing an estimate of the number of patients needing to be treated with NBPs to prevent one additional case of ADRD.

### 2.8 Additional analysis

Subgroup analyses were conducted to investigate potential modifying effects by sex and fracture type. The time‐dependent PS matching and subsequent analysis were repeated within each subgroup. A likelihood ratio test was performed by comparing Cox models with and without an interaction term for exposure and the subgroup variable to test for the modifying effect across subgroups.

Sensitivity analyses were performed to assess the robustness of the findings. First, similar to the “per‐protocol” analysis in RCTs, we censored patients at the time they switched anti‐osteoporosis treatments. While our primary analysis, which did not account for treatment switching, reflects the effectiveness of treatment in real‐world conditions, this “per‐protocol” approach provides a more precise estimate of the treatment effect under ideal adherence. Second, we extended the 6‐month latency period to 12 months to evaluate whether the association would change.

All statistical analyses were conducted using R (version 4.3.1). The cause‐specific Cox model was fitted using the “coxph” function from the survival package. CIFs were estimated using the “cuminc” function from the cmprsk package. The clustered Fine‐Gray model was fitted using the “crrc” function from the crrSC package. A two‐sided *p*‐value < 0.05 was considered statistically significant.[Table alz70503-tbl-0001], [Table alz70503-tbl-0002]

## 3 RESULTS

**FIGURE 1 alz70503-fig-0001:**
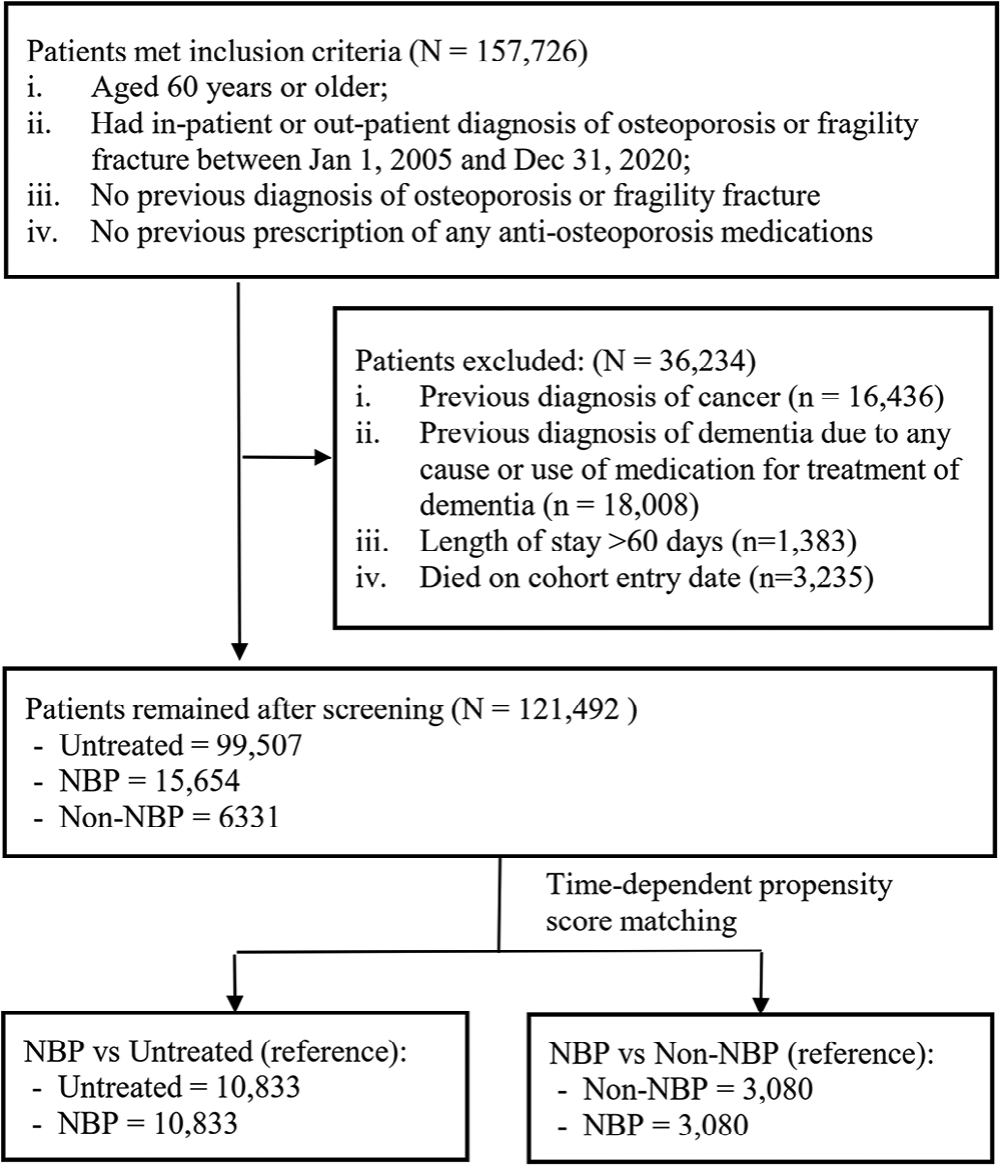
Cohort screening flowchart.

We identified 157,726 patients who met the inclusion criteria and 121,492 patients remained in the cohort after screening (Figure 1). The mean ± SD age of the cohort was 77.3 ± 9.7 years, with the majority (72.5%) being women. Within the first year following diagnosis, 14,822 (12.2%) patients initiated anti‐osteoporosis medication treatment, with 10,856 patients receiving NBPs and 3966 receiving non‐NBP. By the end of 2020, a total of 21,985 (18.1%) patients used the medications (NBP = 15,654; non‐NBP = 6331).

We matched 10,833 pairs of NBP‐exposed and untreated patients, and 3080 pairs of NBP‐exposed and non‐NBP‐exposed patients. The propensity score distribution (SFigure 2) and all the covariates were balanced between the groups (ASD < 0.1, SFigure 3) The baseline characteristics of the cohorts before and after matching are presented in Table 1 and Table 2, respectively. The median follow‐up times were 3.9 years (interquartile range [IQR] = 2.0–6.9) for the matched NBP‐vs‐untreated cohort, and 3.4 years (IQR = 1.9–6.0) for the matched NBP‐vs‐non‐NBP cohort.

**TABLE 1 alz70503-tbl-0001:** Baseline characteristics of the cohorts before propensity score matching.

Parameter	Untreated	Non‐NBP‐exposed	NBP‐exposed
*N*	99507	6331	15654
Exposure on anti‐osteoporosis medication, *n* (%)			
Alendronate	–	–	12463 (79.6)
Ibandronate	–	–	1056 (6.7)
Risedronate	–	–	329 (2.1)
Zoledronate	–	–	1806 (11.5)
Denosumab	–	1989 (31.4)	
Salcatonin	–	3667 (57.9)	
Strontium ranelate	–	421 (6.6)	
Teriparatide	–	254 (4.0)	
Female, *n* (%)	70194 (70.5)	5196 (82.1)	12730 (81.3)
Age, mean (SD)	77.2 (9.9)	78.67 (8.45)	77.2 (8.5)
Fracture type, *n* (%)			
Hip fracture	39382 (39.6)	1837 (29.0)	8332 (53.2)
Wrist fracture	31818 (32.0)	775 (12.2)	2205 (14.1)
Humerus fracture	13207 (13.3)	411 (6.5)	937 (6.0)
Spine fracture	6385 (6.4)	1636 (25.8)	1144 (7.3)
Osteoporosis	7062 (7.1)	1529 (24.2)	2645 (16.9)
Multiple fractures	1653 (1.7)	143 (2.3)	391 (2.5)
Calendar year on cohort entry date, *n* (%)			
2005	5714 (5.7)	304 (4.8)	563 (3.6)
2006	5489 (5.5)	301 (4.8)	632 (4.0)
2007	5298 (5.3)	267 (4.2)	804 (5.1)
2008	5610 (5.6)	337 (5.3)	901 (5.8)
2009	5410 (5.4)	313 (4.9)	1132 (7.2)
2010	5684 (5.7)	348 (5.5)	1138 (7.3)
2011	5660 (5.7)	348 (5.5)	1006 (6.4)
2012	5935 (6.0)	321 (5.1)	897 (5.7)
2013	6252 (6.3)	437 (6.9)	1008 (6.4)
2014	6297 (6.3)	424 (6.7)	1130 (7.2)
2015	6281 (6.3)	437 (6.9)	989 (6.3)
2016	6636 (6.7)	487 (7.7)	1030 (6.6)
2017	6541 (6.6)	520 (8.2)	1098 (7.0)
2018	6616 (6.6)	533 (8.4)	1187 (7.6)
2019	9472 (9.5)	525 (8.3)	1243 (7.9)
2020	6612 (6.6)	429 (6.8)	896 (5.7)
Nursing home residency, *n* (%)	10500 (10.6)	379 (6.0)	834 (5.3)
Medical history, *n* (%)			
Coronary heart disease	10438 (10.5)	738 (11.7)	1446 (9.2)
Congestive heart failure	7301 (7.3)	478 (7.6)	816 (5.2)
Cerebrovascular disease	12707 (12.8)	725 (11.5)	1753 (11.2)
Hypertensive disease	34272 (34.4)	2323 (36.7)	5390 (34.4)
Arrhythmia and conduction disorders	9885 (9.9)	715 (11.3)	1358 (8.7)
Chronic renal disease	4420 (4.4)	276 (4.4)	242 (1.5)
Liver disease	1929 (1.9)	106 (1.7)	290 (1.9)
Chronic pulmonary disease	7748 (7.8)	619 (9.8)	1271 (8.1)
Diabetes	17262 (17.3)	1198 (18.9)	2658 (17.0)
Thyroid disorders	2496 (2.5)	241 (3.8)	476 (3.0)
Obesity	863 (0.9)	41 (0.6)	121 (0.8)
Rheumatic disease	668 (0.7)	87 (1.4)	458 (2.9)
Mental disorders	6969 (7.0)	483 (7.6)	1070 (6.8)
Fall	14267 (14.3)	982 (15.5)	2348 (15.0)
Use of medication in 30 days prior, *n* (%)			
Proton pump inhibitors	13480 (13.5)	1079 (17.0)	2176 (13.9)
Digoxin	2161 (2.2)	115 (1.8)	278 (1.8)
Loop diuretics	11277 (11.3)	774 (12.2)	1486 (9.5)
Other diuretics	6061 (6.1)	444 (7.0)	1068 (6.8)
Anti‐arrhythmics class I and II	1284 (1.3)	86 (1.4)	183 (1.2)
Beta blockers	21928 (22.0)	1488 (23.5)	3438 (22.0)
Angiotensin receptor blocker/ angiotensin converting enzyme inhibitor/ renin inhibitor	24188 (24.3)	1745 (27.6)	3999 (25.5)
Nitrates	8651 (8.7)	630 (10.0)	1189 (7.6)
Calcium channel blockers	42409 (42.6)	2775 (43.8)	6696 (42.8)
Peripheral vasodilators	581 (0.6)	28 (0.4)	81 (0.5)
Anticoagulants	4618 (4.6)	327 (5.2)	824 (5.3)
Platelet inhibitors	23914 (24.0)	1644 (26.0)	3573 (22.8)
Lipid regulating drugs (statins)	23651 (23.8)	1824 (28.8)	4123 (26.3)
Lipid regulating drugs (non‐statins)	1207 (1.2)	81 (1.3)	171 (1.1)
Antipsychotics	3676 (3.7)	154 (2.4)	424 (2.7)
Antidepressants	6074 (6.1)	502 (7.9)	1033 (6.6)
Anti‐Parkinson drugs	3160 (3.2)	179 (2.8)	555 (3.5)
Antidiabetic drugs	21296 (21.4)	1407 (22.2)	3316 (21.2)
Oral corticosteroids	2830 (2.8)	351 (5.5)	778 (5.0)
Non‐steroidal anti‐inflammatory drugs	10695 (10.7)	1149 (18.1)	2028 (13.0)

Abbreviation: NBP, nitrogen‐containing bisphosphonate.

**TABLE 2 alz70503-tbl-0002:** Baseline characteristics of the cohorts after propensity score matching.

Parameter	NBP‐exposed versus Untreated		NBP‐exposed versus non‐NBP‐exposed	
	Untreated	NBP‐exposed	Non‐NBP‐exposed	NBP‐exposed
*N*	10833	10833	3080	3080
Exposure on anti‐osteoporosis medication, *n* (%)				
Alendronate	–	8706 (80.4)	–	2455 (79.7)
Ibandronate	–	783 (7.2)	–	180 (5.8)
Risedronate	–	234 (2.2)	–	78 (2.5)
Zoledronate	–	1110 (10.2)	–	367 (11.9)
Denosumab	–	–	1226 (39.8)	–
Salcatonin	–	–	1407 (45.7)	–
Strontium ranelate	–	–	287 (9.3)	–
Teriparatide	–	–	160 (5.2)	–
Female, *n* (%)	8821 (81.4)	8821 (81.4)	2671 (86.7)	2671 (86.7)
Age, mean (SD)	77.1 (8.4)	77.0 (8.4)	78.32 (8.24)	78.00 (8.09)
Fracture type, *n* (%)				
Hip fracture	6000 (55.4)	6000 (55.4)	1110 (36.0)	1110 (36.0)
Wrist fracture	1742 (16.1)	1742 (16.1)	411 (13.3)	411 (13.3)
Humerus fracture	721 (6.7)	721 (6.7)	166 (5.4)	166 (5.4)
Spine fracture	861 (7.9)	861 (7.9)	426 (13.8)	426 (13.8)
Osteoporosis	1254 (11.6)	1254 (11.6)	916 (29.7)	916 (29.7)
Multiple fractures	255 (2.4)	255 (2.4)	51 (1.7)	51 (1.7)
Calender year on cohort entry date, *n* (%)				
2005	402 (3.7)	402 (3.7)	125 (4.1)	125 (4.1)
2006	436 (4.0)	436 (4.0)	125 (4.1)	125 (4.1)
2007	581 (5.4)	581 (5.4)	120 (3.9)	120 (3.9)
2008	643 (5.9)	643 (5.9)	177 (5.7)	177 (5.7)
2009	813 (7.5)	813 (7.5)	147 (4.8)	147 (4.8)
2010	816 (7.5)	816 (7.5)	187 (6.1)	187 (6.1)
2011	728 (6.7)	728 (6.7)	161 (5.2)	161 (5.2)
2012	645 (6.0)	645 (6.0)	158 (5.1)	158 (5.1)
2013	727 (6.7)	727 (6.7)	195 (6.3)	195 (6.3)
2014	827 (7.6)	827 (7.6)	204 (6.6)	204 (6.6)
2015	695 (6.4)	695 (6.4)	224 (7.3)	224 (7.3)
2016	763 (7.0)	763 (7.0)	250 (8.1)	250 (8.1)
2017	780 (7.2)	780 (7.2)	270 (8.8)	270 (8.8)
2018	850 (7.8)	850 (7.8)	307 (10.0)	307 (10.0)
2019	874 (8.1)	874 (8.1)	307 (10.0)	307 (10.0)
2020	253 (2.3)	253 (2.3)	123 (4.0)	123 (4.0)
Nursing home residency, *n* (%)	1244 (11.5)	478 (4.4)	170 (5.5)	131 (4.3)
Medical history, *n* (%)				
Coronary heart disease	1322 (12.2)	958 (8.8)	337 (10.9)	306 (9.9)
Congestive heart failure	987 (9.1)	505 (4.7)	205 (6.7)	146 (4.7)
Cerebrovascular disease	1672 (15.4)	1207 (11.1)	333 (10.8)	346 (11.2)
Hypertensive disease	4167 (38.5)	3598 (33.2)	1108 (36.0)	1062 (34.5)
Arrhythmia and conduction disorders	1172 (10.8)	924 (8.5)	313 (10.2)	291 (9.4)
Chronic renal disease	552 (5.1)	133 (1.2)	150 (4.9)	45 (1.5)
Liver disease	224 (2.1)	189 (1.7)	56 (1.8)	61 (2.0)
Chronic pulmonary disease	718 (6.6)	847 (7.8)	254 (8.2)	247 (8.0)
Diabetes	2332 (21.5)	1819 (16.8)	585 (19.0)	482 (15.6)
Thyroid disorders	296 (2.7)	333 (3.1)	114 (3.7)	93 (3.0)
Obesity	123 (1.1)	65 (0.6)	24 (0.8)	21 (0.7)
Rheumatic disease	67 (0.6)	136 (1.3)	46 (1.5)	109 (3.5)
Mental disorders	1011 (9.3)	692 (6.4)	221 (7.2)	208 (6.8)
Fall	1551 (14.3)	1606 (14.8)	464 (15.1)	501 (16.3)
Use of medication in 30 days prior, *n* (%)				
Proton pump inhibitors	1461 (13.5)	1449 (13.4)	475 (15.4)	403 (13.1)
Digoxin	293 (2.7)	174 (1.6)	42 (1.4)	42 (1.4)
Loop diuretics	1508 (13.9)	959 (8.9)	396 (12.9)	272 (8.8)
Other diuretics	732 (6.8)	749 (6.9)	216 (7.0)	190 (6.2)
Anti‐arrhythmics class I and II	165 (1.5)	112 (1.0)	46 (1.5)	29 (0.9)
Beta blockers	3027 (27.9)	2351 (21.7)	742 (24.1)	659 (21.4)
Angiotensin receptor blocker/ angiotensin converting enzyme inhibitor/ renin inhibitor	2882 (26.6)	2776 (25.6)	863 (28.0)	781 (25.4)
Nitrates	1114 (10.3)	806 (7.4)	278 (9.0)	249 (8.1)
Calcium channel blockers	5039 (46.5)	4594 (42.4)	1342 (43.6)	1362 (44.2)
Peripheral vasodilators	99 (0.9)	48 (0.4)	13 (0.4)	11 (0.4)
Anticoagulants	657 (6.1)	550 (5.1)	140 (4.5)	159 (5.2)
Platelet inhibitors	3088 (28.5)	2429 (22.4)	794 (25.8)	724 (23.5)
Lipid regulating drugs (statins)	2742 (25.3)	2895 (26.7)	858 (27.9)	894 (29.0)
Lipid regulating drugs (non‐statins)	151 (1.4)	120 (1.1)	40 (1.3)	32 (1.0)
Antipsychotics	603 (5.6)	230 (2.1)	71 (2.3)	71 (2.3)
Antidepressants	808 (7.5)	678 (6.3)	233 (7.6)	207 (6.7)
Anti‐Parkinson drugs	431 (4.0)	379 (3.5)	84 (2.7)	111 (3.6)
Antidiabetic drugs	2962 (27.3)	2287 (21.1)	704 (22.9)	590 (19.2)
Oral corticosteroids	317 (2.9)	397 (3.7)	152 (4.9)	167 (5.4)
Non‐steroidal anti‐inflammatory drugs	1049 (9.7)	1389 (12.8)	505 (16.4)	435 (14.1)

Abbreviation: NBP, nitrogen‐containing bisphosphonate.

**FIGURE 2:**
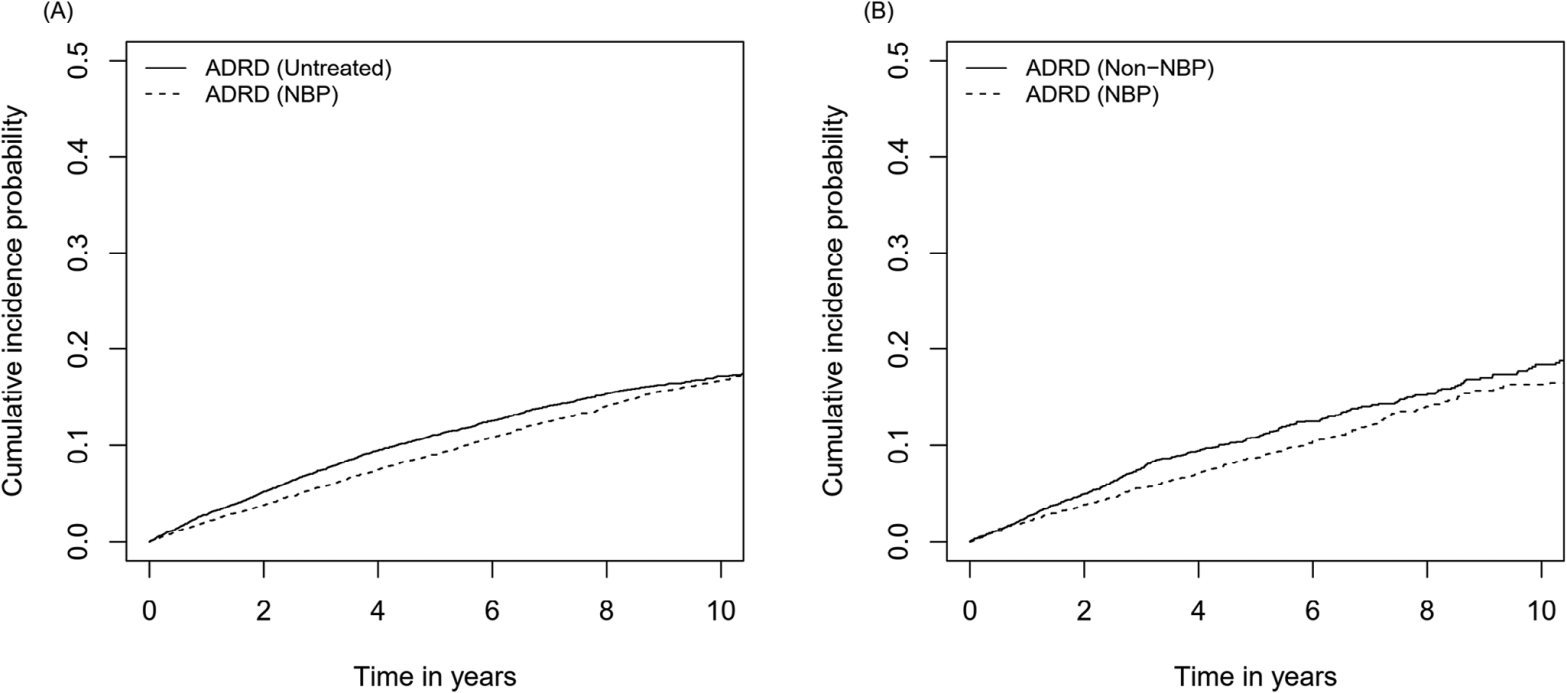
Cumulative incidence function for ADRD in the matched cohort. (A) NBP versus Untreated. A statistically significant lower cumulative incidence of ADRD was observed in NBP‐exposed patients compared to untreated patients (*p* = 0.004 in clustered Fine‐Gray model). (B) NBP vs. non‐NBP. A statistically significant lower cumulative incidence of ADRD was observed in NBP‐exposed patients compared to non‐NBP‐exposed patients (*p* = 0.02 in clustered Fine‐Gray model). ADRD, Alzheimer's disease and related dementia; NBP, nitrogen‐containing bisphosphonates.

The estimated CIFs for ADRD in the exposure groups are described in Figure 2. A statistically significant lower incidence of ADRD was observed in the NBP‐exposed group compared to both the untreated group (*p* = 0.004) and the non‐NBP‐exposed group (*p* = 0.02). The use of NBPs was associated with a reduced risk of ADRD compared to the untreated group (HR 0.84, 95% CI 0.78‐0.90, *p* < 0.001) and non‐NBP‐exposed group (HR 0.76, 95% CI 0.66–0.89, *p* < 0.001) (Table 3). Similar results were obtained in sensitivity analyses using the per‐protocol approach and a 12‐month latency period for outcome (Table 4).

**TABLE 3 alz70503-tbl-0003:** Association of NBP use with the risk of Alzheimer's disease and related dementia.

Analysis	Exposure	*N*	Event	Incidence per 100 person‐years	Median follow‐up time (IQR), year	HR (95% CI)	*p*‐value
NBP versus untreated	Untreated	10833	1350	2.71	3.63 (1.9–6.62)	1 [reference]	
	NBP	10833	1230	2.27	4.11 (2.2–7.14)	0.84 (0.78–0.90)	<0.001
NBP versus non‐NBP	Non‐NBP	3080	352	2.82	3.14 (1.7–5.58)	1 [reference]	
	NBP	3080	305	2.16	3.71 (2.05–6.43)	0.76 (0.66–0.89)	<0.001

Abbreviations: CI, confidence interval; HR, hazard ratio; IQR, interquartile range; NBP, nitrogen‐containing bisphosphonates.

**TABLE 4 alz70503-tbl-0004:** Sensitivity analysis on the association of NBP use with the risk of Alzheimer's disease and related dementia.

Sensitivity analysis	Exposure	*N*	Event	Incidence per 100 person‐years	Median follow‐up time (IQR), year	HR (95% CI)	*p*‐value
Per‐protocol approach	Untreated	10833	1334	2.71	3.59 (1.88–6.56)	1 [reference]	
	NBP	10833	1117	2.25	3.69 (1.93–6.61)	0.83 (0.77–0.90)	<0.001
	Non‐NBP	3080	304	2.96	2.53 (1.39–4.53)	1 [reference]	
	NBP	3080	287	2.22	3.39 (1.85–5.83)	0.74 (0.63–0.87)	<0.001
12‐month latency period	Untreated	9400	1100	2.59	3.63 (1.85–6.51)	1 [reference]	
	NBP	9400	1030	2.26	4.03 (2.1–6.98)	0.87 (0.8–0.94)	<0.001
	Non‐NBP	2498	282	2.89	2.96 (1.63–5.46)	1 [reference]	
	NBP	2498	247	2.28	3.48 (1.94–5.93)	0.79 (0.67–0.93)	0.005

Abbreviations: CI, confidence interval; HR, hazard ratio; IQR, interquartile range; NBP, nitrogen‐containing bisphosphonates.

When comparing NBP‐exposed group to untreated group, the ARDs at 1, 3 and 5 years were 0.007, 0.018, and 0.021, resulting in the NNT of 133, 56, and 48, respectively. Similarly, when comparing NBP‐exposed group to non‐NBP‐exposed group, the ARDs at 1, 3, and 5 years were 0.005, 0.020, and 0.021, resulting in the NNT of 205, 50, and 47, respectively.

**FIGURE 3 alz70503-fig-0003:**
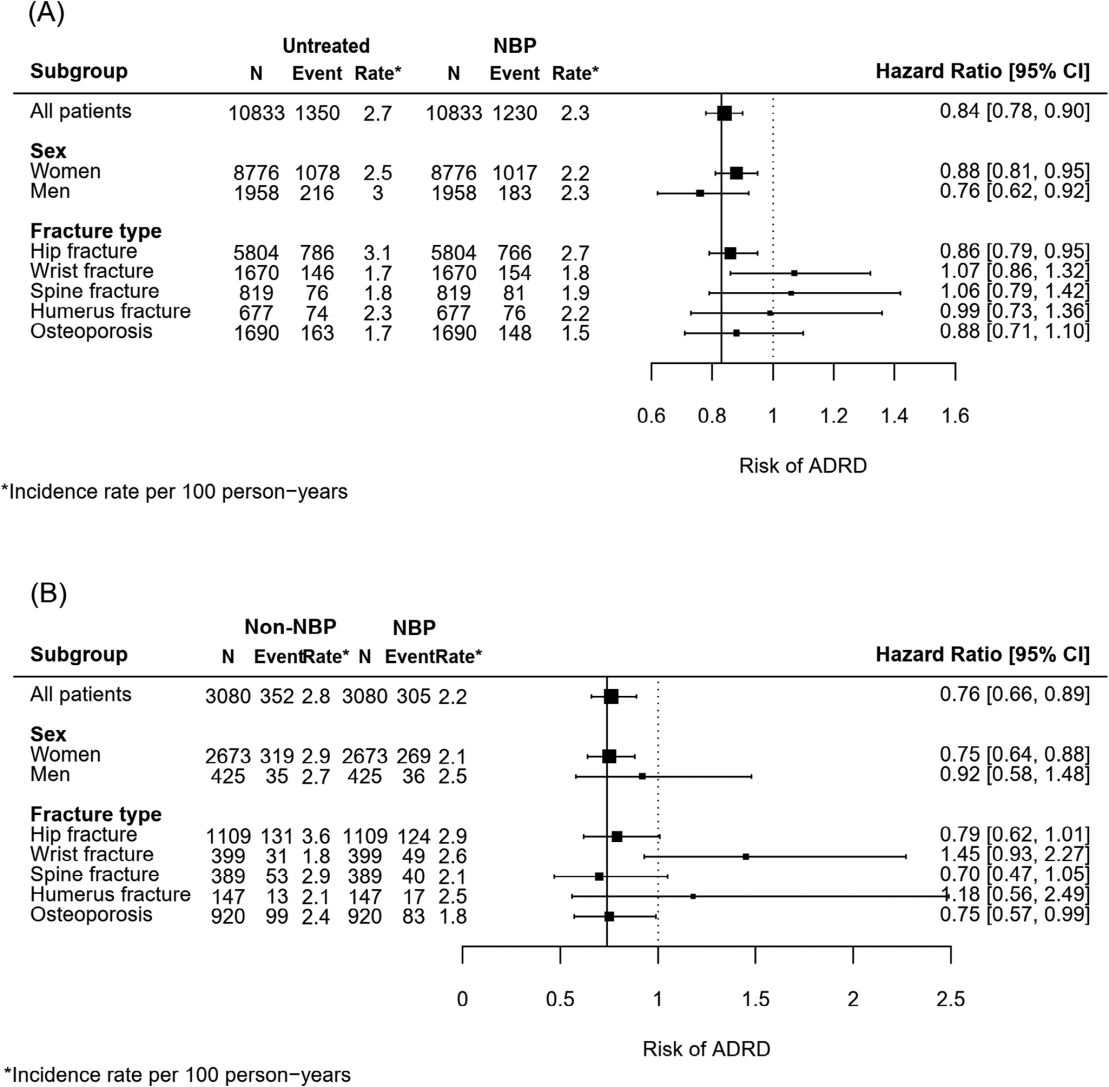
Subgroup analysis by sex and fracture type. (A) NBP vs. Untreated. No statistically significant differences across sex (*p* = 0.19) and fracture type (*p* = 0.39) was shown by likelihood ratio test. (B) NBP vs. non‐NBP. No statistically significant differences across sex (*p* = 0.08) and fracture type (*p* = 0.38) was shown by likelihood ratio test. NBP, nitrogen‐containing bisphosphonates.

The subgroup analysis in the matched NBP‐vs‐untreated cohort showed that the association remained statistically significant only in women (HR 0.88, 95% CI 0.81–0.95, *p* = 0.002), men (HR 0.76, 95% CI 0.62–0.92, *p* = 0.006), and patients with hip fracture (HR 0.86, 95% CI 0.79–0.95, *p* = 0.003) (Figure 3A). Nonetheless, the likelihood ratio test did not indicate a modifying effect (*p* = 0.19 for sex; *p* = 0.39 for fracture type). In the matched NBP‐vs‐non‐NBP cohort, the association remained statistically significant only in women (HR 0.75, 95% CI 0.64–0.88, *p* < 0.001), and patients with osteoporosis (HR 0.75, 95% CI 0.57–0.99, *p* = 0.04) (Figure 3B). Similarly, no modifying effects were found for the subgroups (*p* = 0.08 for sex; *p* = 0.38 for fracture type).

## 4 DISCUSSION

We conducted a population‐based cohort study to investigate the association between the use of NBPs and the risk of ADRD in over 157,000 patients with osteoporosis or fragility fractures. Our findings suggest that the use of NBPs is associated with a reduced risk of ADRD, compared to both non‐users and active comparators. This association remained significant in sensitivity analyses, providing robust evidence for the potential protective effect of NBPs on the risk of dementia.[Table alz70503-tbl-0003], [Table alz70503-tbl-0004]

Our results were consistent with a previous study from Taiwan, which also reported a lower risk of dementia (HR 0.73, 95% CI 0.63–0.84, *p* < 0.001) in patients with osteoporosis or fragility fractures receiving bisphosphonates, compared to those without treatment.^40^ However, the Taiwan study was not primarily designed to evaluate the effect of bisphosphonates and, thus, did not address some common and key biases such as immortal time bias and confounding by indication when patients without treatment are used as comparators. In our study, we used both untreated and active comparators as controls with careful study design and sensitivity analyses to address these potential biases, providing more robust evidence regarding the protective effect of NBPs on dementia risk.[Fig alz70503-fig-0003]

In subgroup analysis, we did not find significant associations in men, and patients with spine fractures, wrist fractures, or humerus fractures. However, the sample size for these subgroups was relatively small resulting in wide CIs. Thus, the null association could be attributed to limited statistical power to detect significance. This is further supported by a post‐hoc power calculation, which indicates that approximately 22,830 and 5630 patients would be needed to achieve 80% of power to detect significant differences when compared to untreated and non‐NBP‐exposed controls (SFigure 4). On the other hand, we cannot rule out the possibility of a sex‐specific association between NBP use and the risk of ADRD. Our previous study^24^ and the Framingham study^41^ have both observed an association between increased bone mineral density (BMD) and reduced risk of dementia in women but not in men, suggesting a sex‐specific association. Future studies with larger sample sizes are warranted to determine sex‐specific associations and the association with other types of fracture.

The neuroprotective effect of NBPs might be explained by its inhibition of FPPS enzyme in the mevalonate pathway, which affects the downstream synthesis of two isoprenoids, FPP and GGPP. Studies suggest that FPP and GGPP are linked to the development of Alzheimer's disease, contributing to the abnormal formation of beta‐amyloid plaques and neurofibrillary tangles, which are composed of hyperphosphorylated tau protein.^42^ Specifically, research has found elevated levels of FPP and GGPP in the brains of patients with Alzheimer's disease compared to cognitively normal individuals,^9^ suggesting a dysregulation of isoprenoid metabolism in Alzheimer's disease that may influence signaling pathways, neuroinflammation, and amyloid‐beta processing. A genetic study has also identified a polymorphic site in the FPPS gene associated with the level of phosphorylated tau protein in the brains of patients with Alzheimer's disease.^10^ In an animal study, the administration of alendronate in high‐fat diet mice improved cognitive function, reduced neuroinflammation, and attenuated amyloid precursor protein processing.^12^ These effects were accompanied by reductions in FPP and GGPP levels and beta‐amyloid deposition in the mice's hippocampus. Similarly, statins, which also target the mevalonate pathway, have been shown to reduce FPP and GGPP levels in the brain, reversing learning and attention deficits in mouse models.^43^ Clinical studies have also reported a lower risk of Alzheimer's disease in statin users.^44^, ^45^, ^46^

NBPs may protect neuronal functions through other mechanisms, such as reducing cholinergic dysfunction, oxidative stress, and neuroinflammation, all of which are recognized as important contributors to Alzheimer's disease.^47^ Animal studies have demonstrated that bisphosphonates can suppress the activity of acetylcholinesterase receptors,^11^ protect muscarinic receptors from free radical damage,^48^ and reduce neuroinflammation biomarkers.^49^

The findings of the current study have important clinical implications. Dementia and osteoporosis share common characteristics and risk factors, and often coexist in the older population.^50^ Patients with dementia are at higher risk of falls and hip fractures,^50^ while osteoporosis and fractures have been identified as independent risk factors for dementia.^40^ Moreover, evidence showed that patients with end‐stage dementia and hip fracture had a poorer recovery from fracture and an almost six‐fold higher risk of mortality compared to cognitively intact individuals.^51^ Given these interconnected relationships, initiating NBPs treatment for patients at high risk of fractures or dementia, such as those carrying the *APOE*4 gene^52^ could be valuable to relieve the healthcare burdens associated with these diseases in the older population.

In addition, it is important to address the issue of under‐treatment of osteoporosis, which is a widespread problem globally.^19^ Previous research by our team has demonstrated that NBP use is associated with reduced risks of cardiovascular disease^20^ and pneumonia.^21^ The current study further highlights the potential neuroprotective effect of NBPs. These additional benefits may serve as an incentive for patients to initiate treatment, ultimately improving treatment rates and overall patient outcomes.

Our study highlights the potential for repurposing NBPs as therapeutic agents for Alzheimer's disease, which is significant considering the limited success of clinical trials for Alzheimer's disease treatment over the past 20 years. Recently, lecanemab^2^ and donanemab,^3^ which are monoclonal antibodies targeting the removal of amyloid β plague, have received full approval from the U.S. FDA. Another monoclonal antibody, remternetug, is undergoing phase III trials with promising findings (NCT05463731). However, it is important to note that these medications only slow down the progression of Alzheimer's disease and do not cure or reverse the cognitive impairment. In contrast, preclinical studies demonstrated that bisphosphonates can reverse impaired cognitive functions in mice models, as aforementioned. Although previous prospective studies failed to demonstrate improvement in cognitive functions accessed by clinical screening tools after the use of zoledronate, these studies were limited by small sample sizes (< 130 subjects) and short follow‐up periods.^53^ ^54^ Our findings, based on the population‐based healthcare data with a long follow‐up period, suggest that further studies are warranted to investigate whether the use of NBPs could improve cognitive functions in patients with cognitive impairment or ADRD.

NBPs is widely used to reduce the risk of skeletal‐related events in patients with cancers, particularly breast, prostate, and lung cancer.^55^ Several observational studies have shown that cancer is associated with reduced risk of ADRD by 15% to 37%.^56^, ^57^, ^58^ While the underlying mechanism is not yet fully understood, our findings suggest a potential role of NBPs in preventing ADRD among cancer patients and warrant further investigation.

This study has several strengths. First, we used a territory‐wide clinical database that captured comprehensive and valid records, providing real‐world evidence on the association between NBPs use and a reduced risk of dementia. This enhances the generalizability and reliability of the findings. In addition, the study was carefully designed to address common biases in pharmacoepidemiological studies. We employed a time‐dependent PS matching to address immortal time bias. Both non‐users and active comparators were used as controls to minimize the confounding by indication and similar results were observed.

However, there are limitations to consider. First, the assessment of cognitive function at baseline was not available in the database. It is possible that patients with poor cognitive function were less likely to receive treatment and had a higher risk of dementia. However, we incorporated a 6‐month latency period to exclude patients with a diagnosis of dementia during this period to minimize the bias. Second, data on severity of osteoporosis and fragility fracture, including BMD and physical functioning, were not available. Patients with a lower BMD (indicating more severe osteoporosis) would be more likely to initiate anti‐osteoporosis treatment. However, studies have shown that low BMD is associated with poorer cognitive function and a higher risk of dementia,^40^ ^59^ ^60^ suggesting that any bias in this regard would likely underestimate, rather than overestimate, the protective treatment effect. While we lacked data on physical functioning in this study, our previous research indicates that treatment decisions among hip fracture patients are unlikely to be confounded by this factor.^20^ Therefore, we believe the overall bias introduced by unmeasured disease severity is minimal. Third, over‐the‐counter medication records, such as vitamin D and calcium supplements, were not captured in the database, which could potentially confound the results. Fourth, we lacked data on several risk factors for dementia, such as air pollution, social isolation, visual loss, and education, as highlighted in the 2024 Lancet Commission report.^61^ While our database can identify ocular comorbidities, including cataract (ICD‐9 366.xx), age‐related macular degeneration (ICD‐9 362.5x), glaucoma (ICD‐9 365.xx), diabetic retinopathy (ICD‐9 250.5x, 362.02), and visual impairment (ICD‐9 369.xx) using ICD‐9 codes, it lacks data on visual acuity necessary for accurate assessment of visual loss. Using ICD‐9 codes, we observed that in the matched cohorts, the prevalence of ocular comorbidities at baseline (excluding those who underwent cataract surgery) were similar across treatment groups (NBP‐exposed vs. untreated: 9.1% vs. 9.7%; NBP‐exposed vs. non‐NBP‐exposed: 9.6% vs. 10.6%). This similarity suggests that any confounding related to visual loss is likely minimal. Lastly, like other observational studies, there may be potential residual confounding.

In conclusion, the use of NBPs among patients with osteoporosis or fragility fractures was associated with a lower risk of ADRD compared to patients who did not use these medications. Further studies are warranted to validate the neuroprotective effect of bisphosphonates. If the findings are validated, it is encouraged to initiate bisphosphonate treatment in patients at high risk of ADRD to improve patient outcomes.

## CONFLICT OF INTEREST STATEMENT

The authors declare no conflicts of interest. Author disclosures are available in the supporting information.

## CONSENT STATEMENT

Consent was not necessary.

## Supporting information



Supporting Information

Supporting Information
