# Supplementary material for: Bisphosphonates and the risk of dementia in patients with osteoporosis or fragility fracture: A population‐based study in Hong Kong
Source: Alzheimers Dement. 2025 Jul 21;21(7):e70503. doi: 10.1002/alz.70503 (PMC12279470; doi:10.1002/alz.70503)
Supplement: Supplementary file 1 — Supporting Information [file ALZ-21-e70503-s002.docx]

**Supplementary Materials**

Bisphosphonates and the risk of dementia in patients with osteoporosis or fragility fracture. A population-based study in Hong Kong

| Supplementary Method | p.2 |
| --- | --- |
| STable 1. Variable coding for study cohort, exposure of interest, and outcome of interest | p.3 |
| STable 2. Variables in the propensity score model | p.5 |
| SFigure 1. Study design schema | p.7 |
| SFigure 2. Distribution of propensity score before and after matching | p. 8 |
| SFigure 3 Covariate balance before and after matching (A) NBP vs Untreated; (B) NBP vs Non-NBP | p.9 |
| SFigure 4. Post-hoc power calculation | p.10 |

**Supplementary Method**

Validation of the diagnostic coding for dementia in CDARS database

The validation was conducted in Queen Mary Hospital, an acute hospital located in the Hong Kong West district. Queen Mary Hospital is a teaching hospital for the Li Ka Shing Faculty of Medicine of The University of Hong Kong as well as a territory-wide tertiary and quaternary referral center for advanced medical service.

Diagnosis of dementia was defined using ICD-9 code 290.xx (Dementias), 294.8x (Other specified mental disorder due to another medical condition), and 331.0x (Alzheimer’s disease). We identified 155,660 patients who had been diagnosed with dementia in Queen Mary Hospital between Jan 1, 2005, and Dec 31, 2020. Given that it is not feasible to review such a large number of patients, we randomly selected a sample of 100 patients for validation. Two researchers independently reviewed the clinical notes written by physicians and validated the cases according to the criteria suggested by the Diagnostic and Statistical Manual of Mental Disorders, Fifth Edition (DSM-5) [1]. Disagreement in the validation was sought by discussion to reach a consensus.

The criteria for major neurocognitive disorder (dementia) are shown as follows:

1. Evidence of significant cognitive decline from a previous level of performance in one or more cognitive domains (complex attention, executive function, learning and memory, language, perceptual-motor, or social cognition) based on:
   1. Concern of the individual, a knowledgeable informant, or the clinician that there has been a significant decline in cognitive function; and
   2. A substantial impairment in cognitive performance, preferably documented by standardized neuropsychological testing or, in its absence, another quantified clinical assessment.
2. The cognitive deficits interfere with independence in everyday activities (i.e., at a minimum, requiring assistance with complex instrumental activities of daily living such as paying bills or managing medications).
3. The cognitive deficits do not occur exclusively in the context of a delirium.
4. The cognitive deficits are not better explained by another mental disorder (e.g., major depressive disorder, schizophrenia).

True positive cases were defined as cases that met all criteria in DSM-5, while false positive cases were cases that did not meet the criteria in DSM-5 or incomplete information to determine the diagnosis. We calculated the positive predictive value (PPV) by dividing the number of true positive cases by the total number of true positives and false positives. The 95% confidence interval was estimated using binomial distribution.

The mean±SD age of the samples was 81.1±6.93 and 60% of the patients were women. The age and sex distribution were similar to the original cohort (mean±SD age = 82.5±8.64, women = 60.4%). After validation, we found that 81 true positive cases and 19 false positive cases; Of 19 false positive cases, 10 cases did not meet the criteria, and 9 cases had incomplete information to determine the diagnosis. The PPV was calculated to be 81% (95% CI 77.1-84.9).

References

1. American Psychiatric Association. Diagnostic and Statistical Manual of Mental Disorders (DSM-5). 5th ed. Arlington, VA: Arlington; 2013.

STable 1. Variable coding for study cohort, exposure of interest, and outcome of interest

| Variable | Coding system | Code | Description |
| --- | --- | --- | --- |
| **Variables in identifying study cohort** | | | |
| Osteoporosis | ICD-9 | 733.0 | Osteoporosis |
| Spine fracture | ICD-9 | 805 | Fracture of vertebral column without mention of spinal cord injury |
| Humerus fracture | ICD-9 | 812 | Fracture of humerus |
| Wrist fracture | ICD-9 | 813  814 | Fracture of radius and ulna  Fracture of carpal bone(s) |
| Hip fracture | ICD-9 | 820 | Fracture of neck of femur |
| Anti-osteoporosis medications | BNF | 6.6.1  6.6.2 | Calcitonin and parathyroid hormone  Bisphosphonates and other drugs affecting bone metabolism |
| **Variables in excluding patients** | | | |
| Cancer | ICD-9 | 140-208 | Malignant Neoplasm |
| Dementia due to any cause | ICD-9 | 290  291.2  292.82  294.1  294.2  294.8  331.0  331.1  331.82 | Dementias  Alcohol-induced persisting dementia  Drug-induced persisting dementia  Dementia in conditions classified elsewhere  Dementia, unspecified  Other persistent mental disorders due to conditions classified elsewhere  Alzheimer's disease  Frontotemporal dementia  Dementia with Lewy bodies |
| Medication for treatment of dementia | BNF | 4.11 | Drugs for dementia |
| **Variable in outcome of interest** | | | |
| Alzheimer’s disease and related dementia (including Alzheimer’s disease, vascular dementia, senile dementia, and dementia with an unspecified cause) | ICD-9 | 290  294.8  331.0 | Dementias  Other persistent mental disorders due to conditions classified elsewhere  Alzheimer's disease |
| **Variables in exposure of interest** | | | |
| Nitrogen-containing bisphosphonate (including alendronate, ibandronate, risedronate, and zoledronate) | Local drug code | ALEN, FOSA, S00174, S00517, S00641, | Alendronate sodium [10mg, 70mg] |
|  |  | IBAN, S00510, S00597, S00532, S00606 | Ibandronic acid [50mg, 150mg, 1mg/ml] |
|  |  | RISE, S00224,  S00110 | Risedronate sodium [5mg, 35mg] |
|  |  | ZOLE02, S00627 | Zoledronic acid (aclasta) [50mcg/ml] |
| Non-Nitrogen-containing bisphosphonate (including denosumab, salcatonin, strontium ranelate, and teriparatide) |  | DENO02, S00944 | Denosumab (prolia) [60mg/ml] |
|  |  | SALC04, SALC05, SALC06 | Salcatonin (calcitonin salmon) nasal spray [50IU/dose, 100IU/dose, 200IU/dose] |
|  |  | STRO03, S00516 | Strontium ranelate [2g/sachet] |
|  |  | TERI, S01098, S00698 | Teriparatide [250mcg/ml] |

STable 2. Variables in the propensity score model

| **Variables** | |
| --- | --- |
| Sex | |
| Age | |
| Calendar year | |
| Fracture type (osteoporosis, hip fracture, humerus fracture, spine fracture, wrist fracture, multiple fractures) | |
| Nursing home residency | |
| **Medical history** | **ICD-9 code** |
| Coronary heart disease | 410, 411, 412, 413, 414, 429.2, 429.71, 429.79 |
| Congestive heart failure | 398.91, 402.01, 402.11, 402.91, 404.01, 404.03, 404.11, 404.13, 404.91, 404.93, 428 |
| Cerebrovascular diseases | 362.34, 430, 431, 432, 433, 434, 435, 436, 437, 438 |
| Hypertensive diseases | 401, 402, 403, 404, 405 |
| Arrhythmia and conduction disorders | 426, 427 |
| Chronic renal disease | 403, 404, 582, 583.0-583.7, 585, 586, 588.0, 590.0, V56 |
| Liver disease (esophageal varices, chronic hepatitis, cirrhosis) | 070.22, 070.23, 070.32, 070.33, 070.44, 070.54, 456.0, 456.1, 456.2, 571, 572.2, 572.3, 572.4, 572.8 |
| Chronic pulmonary disease | 490, 491, 492, 493, 494, 495, 496, 500, 501, 502, 503, 504, 505, 506.4, 508.1, 508.8 |
| Diabetes | 250 |
| Overweight and obesity | 278.0 |
| Thyroid disorders | 242, 243, 244 |
| Rheumatic disease | 446.5, 710.0, 710.1, 710.2, 710.3, 710.4, 714.0, 714.1, 714.2, 714.8, 725 |
| Mental disorders (depression, anxiety, adjustment disorder, schizophrenia, bipolar disorder) | 293.83, 293.84, 296.4, 295, 296.2, 296.3, 296.5, 296.6, 296.7, 296.99, 300.00, 300.01, 300.02, 300.09, 300.21, 300.22, 300.23, 300.29, 300.4, 309, 311, 313.23 |
| Fall | E880, E881, E882, E883, E884, E885, E886, E888, V15.88 |
| **Use of medication in 30 days prior** | **BNF code** |
| Proton-pump inhibitors | BNF 1.3.5 |
| Digoxin | BNF 2.1.1 |
| Loop diuretics | BNF 2.2.2 |
| Other diuretics | BNF 2.2.1, 2.2.3, 2.2.4, 2.2.5 |
| Anti-arrhythmics class I and II | BNF 2.3.2 |
| Beta-blockers | BNF 2.4 |
| Angiotensin II receptor blockers / Angiotensin-converting enzyme-I | BNF 2.5.5.1, 2.5.5.2 /2.5.5.3 |
| Nitrates | BNF 2.6.1 |
| Calcium channel blockers | BNF 2.6.2 |
| Peripheral vasodilators | BNF 2.6.4 |
| Anticoagulants | BNF 2.8 |
| Platelet inhibitors | BNF 2.9 |
| Lipid regulating drugs (Statins) | BNF 2.12 (included atorvastatin, fluvastatin, lovastatin, pravastatin, rosuvastatin, and simvastatin) |
| Lipid regulating drugs (Non-statins) | BNF 2.12 (included non-statins drugs) |
| Antipsychotics | BNF 4.2 |
| Antidepressants | BNF 4.3 |
| Anti-Parkinson drugs | BNF 4.9 |
| Antidiabetic drugs | BNF 6.1.1, 6.1.2 |
| Oral corticosteroids | BNF 6.3 *only oral/injection/parenteral |
| Nonsteroidal anti-inflammatory drugs | BNF 10.1.1 |


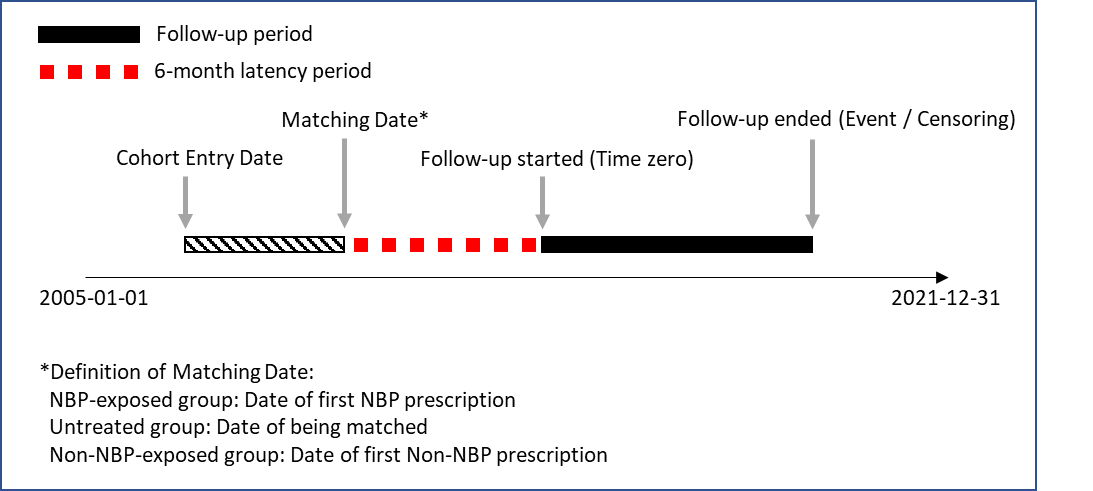


SFigure 1. Study design schema


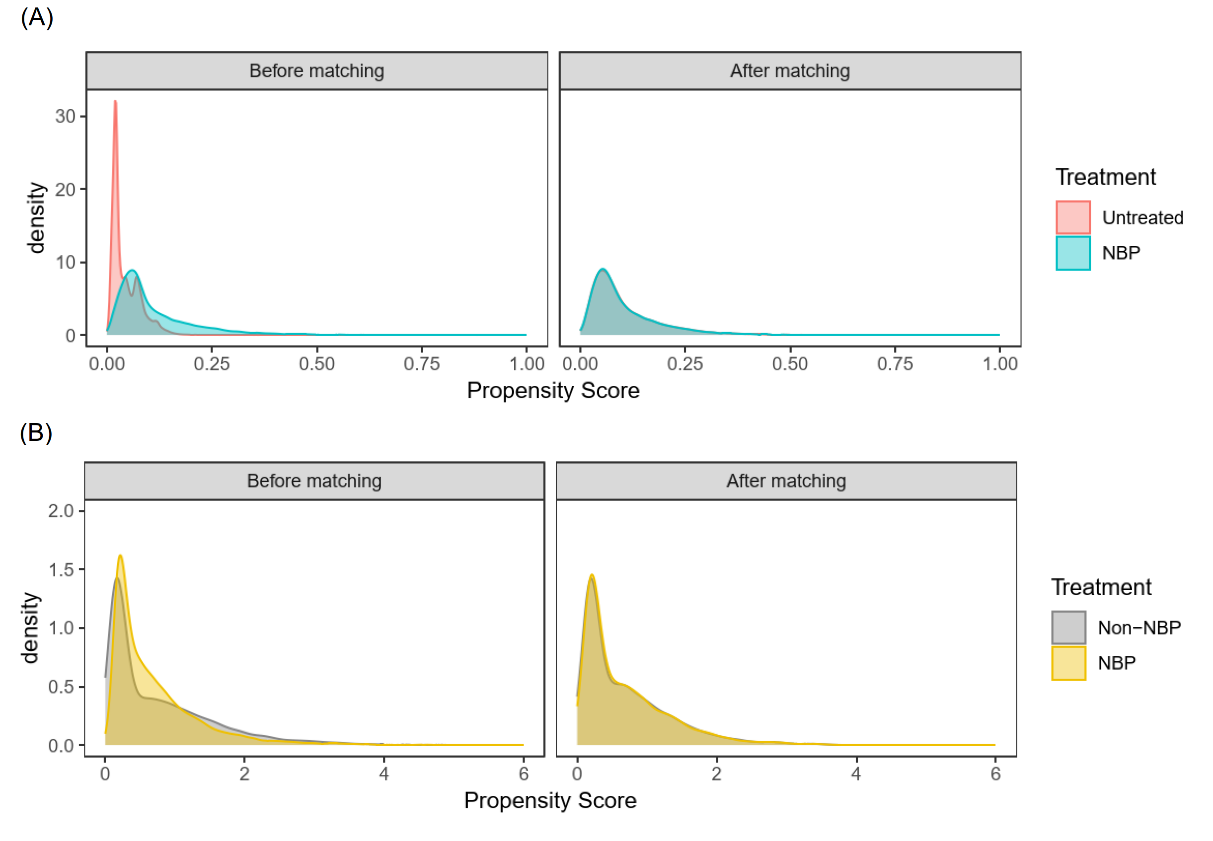


SFigure 2. Distribution of propensity score before and after matching (A) NBP vs Untreated; (B) NBP vs Non-NBP

| (A) | (B) |
| --- | --- |
| 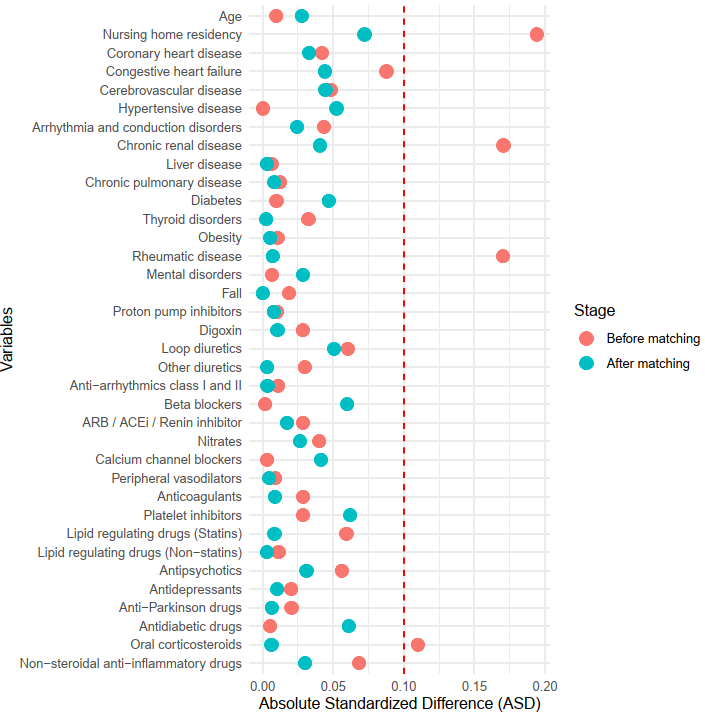 | 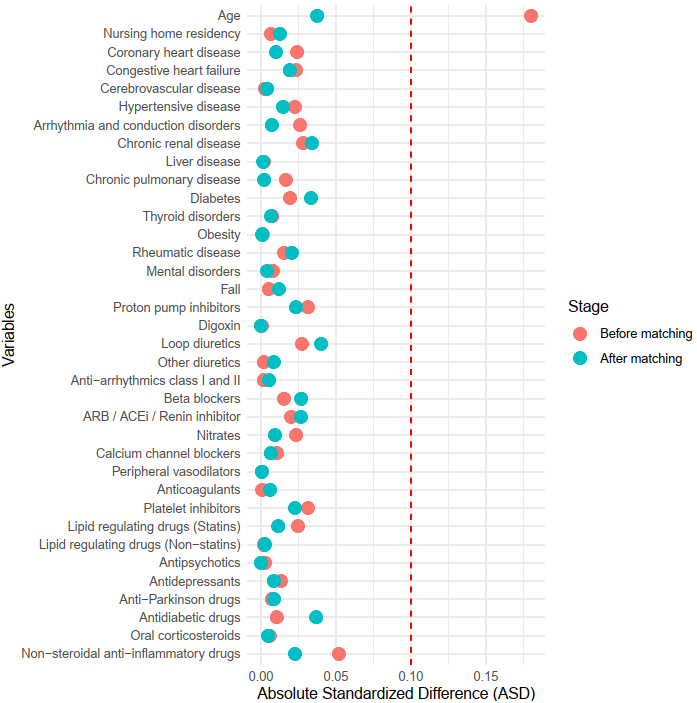 |

SFigure3 Covariate balance before and after matching (A) NBP vs Untreated; (B) NBP vs Non-NBP.

| a) NBP vs Untreated  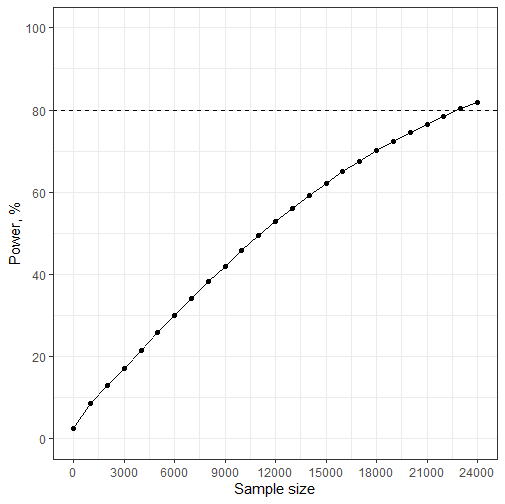 | b) NBP vs non-NBP  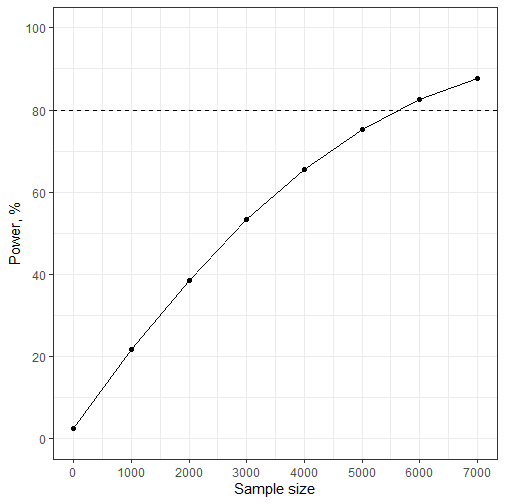 |
| --- | --- |

SFigure 4. Post-hoc power calculation. R package “powerSurvEpi” was used to calculate the power a) NBP vs untreated, assuming postulated hazard ratio= 0.84, treatment proportion=13%, disease prevalence=10%, and alpha=0.05; b) NBP vs non-NBP, assuming postulated hazard ratio= 0.76, treatment proportion=71%, disease prevalence=9%, and alpha=0.05.
